# Heroin-Induced Acute Pancreatitis

**DOI:** 10.7759/cureus.15470

**Published:** 2021-06-05

**Authors:** Camelia Ciobanu, Raja Shekar Jadav, Ana Colon Ramos, Harry G Sequeira Gross, Carlos Brazzarola

**Affiliations:** 1 Medicine, St. Barnabas Hospital, Bronx, USA; 2 Cardiovascular Disease, Mayo Clinic, Rochester, USA; 3 Internal Medicine, St. Barnabas Hospital, Bronx, USA; 4 Internal Medicine, St. Barnabas Hospital (SBH) Health System, Bronx, USA

**Keywords:** heroin induced pancreatitis, acute pancreatitis, drug induced pancreatitis, heroin use disorder, opioids

## Abstract

Heroin-induced pancreatitis (HIP) is rare with only a few cases reported previously in the literature and the pathophysiology mechanism is yet to be investigated. We present two cases of acute pancreatitis (AP) in the setting of acute heroin (diacetylmorphine) intoxication. Both patients presented with nausea, vomiting and severe abdominal pain after intranasal heroin use. On laboratory analysis were found to have elevated serum lipase, positive urine toxicology for opioids, without any other obvious causes for AP. Both patients had a full recovery with supportive treatment. As a general approach, drug-induced pancreatitis is a diagnosis of exclusion and a high index of suspicion is required when the most common etiologies are ruled out.

## Introduction

Drug-induced pancreatitis is relatively rare and often poses a diagnostic challenge for clinicians as more than 30 drugs are implicated in the definitive causality according to the World Health Organization, and opiates being one of them [1]. To date, there is limited data about the accurate incidence and prevalence of drug-induced pancreatitis, and it is estimated to represent 1.4%-5.3% [2-4].

Opiates such as codeine, morphine are known to cause elevated serum amylase levels, pancreatic and biliary duct dilatation with spasm of the sphincter of Oddi [5-9]; however, the exact pathophysiology is yet to be elucidated. Heroin-induced pancreatitis (HIP) is a rare entity with only two reported cases in the literature [5,10]. We present two cases of acute pancreatitis (AP) in the setting of acute heroin (diacetylmorphine) intoxication.

## Case presentation

Case 1

A 50-year-old male with past medical history of hypertension, heroin use disorder presented to the emergency department (ED) with nausea, vomiting, and abdominal pain started after using 2-3 grams of intranasal heroin, daily, for the past two weeks. Vital signs on presentation were: temperature 38℃, heart rate 100 beats per minute, blood pressure 157/102 mmHg, respiratory rate 20 breaths/min, and oxygen saturation 95% on room air. Physical examination was pertinent for moderate diffuse abdominal tenderness to palpation. Laboratory data revealed leukocytosis with left shift, acute kidney injury with serum creatinine 1.3 mg/dL, elevated liver enzymes with alanine transaminase/aspartate transaminase (ALT/AST) 45/45 IU/L, high serum lipase 389 U/L (normal: 22-51 U/L), negative blood alcohol, calcium 10.4 mg/dL (see Table 1), triglyceride levels 105 mg/dL and negative viral hepatitis markers. Serum 10 drug screen (that tests for codeine, morphine, hydromorphone, hydrocodone, etc.) and urine toxicology were positive for opiates. He denied any history of previous similar episodes, alcohol or medication use, trauma, autoimmune diseases. Abdomen/pelvis computed tomography (CT) was negative for pancreatic edema, ductal dilatation, or gallstones. The patient was admitted for acute pancreatitis secondary to heroin use and was managed with supportive care such as intravenous fluids, antiemetics, and methadone taper with the resolution of symptoms.

**Table 1:** Physiologic parameters over time. *Note: Within the first 48 hours, both patients showed the presence of 1 Ranson’s prognostic criteria consistent with mild acute pancreatitis.

Physiologic parameters over time*				
	Case 1		Case 2	
Parameters	On arrival	48 hours	On arrival	48 hours
Sodium (mEq/L)	139	139	137	138
Potassium (mEq/L)	3.4	3.1	3.8	3.9
Calcium (mg/dL)	10.4	9.1	9.7	9.0
Serum glucose (mg/dL)	143	105	106	44
Urea nitrogen (mg/dL)	19	16	10	8
Serum creatinine (mg/dL)	1.3	1.1	0.9	0.8
Albumin (g/dL)	4.6	4.0	3.9	3.4
Alanine transaminase (U/L)	45	31	36	56
Aspartate aminotransferase (U/L)	45	26	51	72
Total bilirubin (mg/dL)	0.8	0.7	1.8	1.9
Lipase (U/L)	389	39	155	-
Hemoglobin (g/dL)	16.2	14.4	12.4	12.7
Hematocrit (%)	46.6	43.2	40.7	40.1
White blood cell (cells/microliter)	17,900	11,800	5,800	5,400

Case 2

A 74-year-old man with medical history of benign prostatic hyperplasia, heroin use disorder on a methadone maintenance program, cholecystectomy came to the ED complaining of severe epigastric abdominal pain, nausea, vomiting that started a couple of hours after taking his daily dose of methadone 50 mg along with one bag of intranasal heroin and self-administration of naloxone. On vital signs, the patient was normotensive, afebrile, heart rate 88 beats/min, and tachypneic 25 breaths per min. Physical examination was remarkable for epigastric tenderness. Laboratory analysis showed normocytic anemia with hemoglobin 12.4 gm/dL, AST 51 IU/L, total bilirubin 1.8 mg/dL, elevated lipase 155 U/L, lactic acidosis 4.4 mmol/L, negative blood alcohol, calcium 9.7 mg/dL (Table 1), and triglyceride levels 89 mg/dL. Urine toxicology was positive for opiates. He denied any history of recent medication use, trauma, alcohol, autoimmune diseases or previous history of similar abdominal pain or pancreatitis. CT abdomen/pelvis showed unremarkable pancreas, absent gallbladder, and no ductal dilatation. He was admitted to the medical floor under the impression of acute pancreatitis secondary to heroin use and was treated conservatively with intravenous fluids, antiemetics with the resolution of symptoms.

## Discussion

Diagnosing acute pancreatitis requires the presence of at least two criteria from the following three: acute onset of epigastric abdominal pain, elevated serum lipase or amylase more than three times of upper limit, and signs of pancreatic inflammation on abdominal imaging studies. Establishing the cause of acute pancreatitis plays a vital role in the management and prevention of recurrences. A thorough medical history and accurate details about recent or active drug use, including recreational drugs, are essential. After ruling out the most common etiologies such as gallstones, alcohol, hypercalcemia, hypertriglyceridemia, anatomic anomalies like pancreatic divisum, and tumors, drugs should always be considered in the differential as one of the culprits. 

To date, there is no universally accepted definition or classification of drug-induced pancreatitis, and all the available information is gathered from case reports or reviews. The latest classification proposed is the definite, probable and possible correlation of a particular medication to cause drug-induced pancreatitis and opioids are categorized as definite causality [1].

In a review by Trivedi et al. [11], all reported cases of drug-induced pancreatitis from 1966 to April 30, 2004, were evaluated and grouped into three classes where class I represented the medications with the strongest evidence of correlation with acute pancreatitis. Opioids along with azathioprine, asparaginase, didanosine, valproic acid, mercaptopurine, mesalamine, estrogen, steroids, sulfasalazine, etc., are categorized as class I. Opioids were described in a total of forty-two cases, including five re-exposure cases and codeine was most commonly identified [7-9,12-15]. An important feature of this [11] publication is that they also looked at commonly prescribed medication involved in the development of drug-induced pancreatitis, and oxycodone was reported in 55 cases [11]. It is highly possible that there are many unreported or underdiagnosed cases, and the numbers are much higher, especially with the broader utilization of opioids in clinical settings for pain management or as recreational drugs. 

Codeine (methyl morphine) is metabolized to morphine via cytochrome P450 2D6 (CYP2D6), a polymorphic gene that has three variations when it comes to O-demethylation, and depending on the gene activity, will generate ultra-rapid metabolizers, extensive metabolizers, and poor metabolizers [14,16]. Ultra-rapid metabolizers will have higher levels of morphine in the serum and urine, higher analgesic effect, along with an increased risk of adverse reactions and complications [14].

Morphine is broadly used for pain management in patients with acute pancreatitis, although on literature review, it is also associated with dysfunction of sphincter Oddi, including elevation in bile duct pressure, a significant increase in contractile amplitude and frequency of the sphincter of Oddi [6,17,18]. In a study performed on mice, the impact of morphine on the progression of acute pancreatitis was analyzed and found to increase pancreatic neutrophilic infiltration and necrosis along with increase gut permeability, bacterial translocation, and bacteremia, which aggravated acute pancreatitis, delayed resolution and regeneration [19]. However, there are no human studies or case reports available to support the above findings. It is yet unclear what is the pathophysiological mechanism by which diacetylmorphine (heroin) produces HIP, and one proposed theory is heroin-induced vasospasm [20], although direct cytotoxic effects of heroin alone or agents used to cut heroin [21] (baking soda/starch, etc.) on pancreatic cells cannot be excluded.

Treatment of drug-induced pancreatitis involves the withdrawal of the offending agent and supportive therapy; thus, an early diagnosis would help in reducing morbidity and length of hospital stay as seen in our patients. Within 24 hours of hospitalization, we were able to rule out the most common causes of AP and in the setting of known exposure to heroin, this was the most appropriate explanation of AP, and our patients did not receive any opioids for pain management. 

Our patients can be categorized as mild AP as per Ranson’s criteria in the setting of intranasal heroin use with negative CT findings, whereas previously reported cases [5,10] had severe AP despite negative imaging in one case [10], and both had the same route of heroin administration. The severity of the disease may depend on the amount and or the route of intake; this remains unclear and needs further research. 

Generally, the subjects with drug-induced pancreatitis have a good prognosis with an uneventful clinical course [4,7] as noted in our patients, albeit in one of HIP described in the literature, the patient expired due to severe complications [10]. Therefore, clinicians must be aware of the most common medications implicated in drug-induced pancreatitis, especially opioids, in the age of rising heroin addiction. 

## Conclusions

AP is a serious condition with potential high morbidity and mortality if it is not diagnosed and treated in a timely manner. Drug-induced pancreatitis is a diagnosis of exclusion. A strong understanding of the drugs associated with its causality is needed, as knowledge of HIP may help clinicians to have a high index of suspicion and establish an early diagnosis. Further studies are necessary to evaluate the underlying mechanism, actual incidence, and the risk of HIP. 
